# A Magnetic-Multiplier-Enabled Hybrid Generator with Frequency Division Operation and High Energy Utilization Efficiency

**DOI:** 10.34133/research.0168

**Published:** 2023-06-09

**Authors:** Jie Chen, Kangjie Wu, Shaokun Gong, Jianchao Wang, Ke Wang, Hengyu Guo

**Affiliations:** ^1^ College of Physics and Electronic Engineering, Chongqing Key Laboratory Photo-electric Functional Materials, Chongqing Normal University, Chongqing 401331, China.; ^2^ State Key Laboratory of Mechanical Transmission, College of Mechanical and Vehicle Engineering, Chongqing University, Chongqing 400044, China.; ^3^ School of Physics, Chongqing University, Chongqing 400044, China.

## Abstract

The hybrid electromagnetic-triboelectric generator (HETG) is a prevalent device for mechanical energy harvesting. However, the energy utilization efficiency of the electromagnetic generator (EMG) is inferior to that of the triboelectric nanogenerator (TENG) at low driving frequencies, which limits the overall efficacy of the HETG. To tackle this issue, a layered hybrid generator consisting of a rotating disk TENG, a magnetic multiplier, and a coil panel is proposed. The magnetic multiplier not only forms the EMG part with its high-speed rotor and the coil panel but also facilitates the EMG to operate at a higher frequency than the TENG through frequency division operation. The systematic parameter optimization of the hybrid generator reveals that the energy utilization efficiency of EMG can be elevated to that of rotating disk TENG. Incorporating a power management circuit, the HETG assumes the responsibility for monitoring the water quality and fishing conditions by collecting low-frequency mechanical energy. The magnetic- multiplier-enabled hybrid generator demonstrated in this work offers a universal frequency division approach to improve the overall outputs of any hybrid generator that collects rotational energy, expanding its practical applications in diverse multifunctional self-powered systems.

## Introduction

Rapid development of the next-generation wearable and autonomous devices intensified the need for a reliable energy supply. Currently, chemical batteries are the primary power source for these electronics, which are plagued by poor battery life, electrolyte leaks, and environmental contamination. Thus, how to collect energy from the ambient environment and convert it into efficient electricity is a hot issue that urgently needs to be addressed for the alternative means of conventional energy supply. Among the various energy sources, mechanical energy is the one that deserves to be investigated in terms of its abundance, accessibility, and ubiquity in surroundings. Harvesters for mechanical energy typically involve in electromagnetic [1,2], triboelectric [3–5], and piezoelectric effects [6–8], where the electromagnetic generator (EMG) and triboelectric nanogenerator (TENG) are 2 most efficient approaches [9–11]. The Faraday’s law of electromagnetic induction-based EMG requests the coordination of coils and magnets, which produces a high output current but low output voltage and excels in high-frequency energy scavenge [12–14]. Through the combination of triboelectrification and electrostatic induction, TENG exhibits superior performance in harvesting low-frequency energy with the advantages of diverse material selection, simple structure, low cost, and light weight [15–17]. Meanwhile, compared to single EMG or TENG device, the operating frequency range is broadened obviously by the introduction of hybrid electromagnetic-triboelectric generator (HETG) [18,19].

Since 2015, HETG has been designed into various structures to apply in biomechanical [20–23], wind [24–27], vibration [28–31], and wave energy harvesting [32–35]. However, the majority of HETGs consist of a simple combination of EMG and TENG operating at the same frequency, which necessitates a driving source with a wide input frequency range or allows only 1 of the 2 components to operate at its appropriate operating frequency. Hu et al. [36] conducted a detailed quantitative comparison between TENG and EMG, which reveals that TENG has a superior energy utilization efficiency compared to EMG at low frequencies. At a frequency of 2.5 Hz, TENG exhibits a remarkable energy utilization efficiency of 75%, whereas EMG yields only 3%. The highest energy utilization efficiency of TENG, reaching 98.84%, is observed at a frequency of 5 Hz. However, as the frequency increases, the energy utilization efficiency of TENG declines, while that of EMG gradually increases. That is, the optimal frequency ranges for energy utilization efficiency differs between TENG and EMG. Consequently, a division frequency strategy is crucial for the 2 components of HETG to operate in their respective frequency domains, thereby enhancing the energy utilization efficiency.

In this work, a layered hybrid generator made by a rotating disk TENG (RTENG), a magnetic multiplier, and a coil panel is designed to realize the division frequency operation of TENG and EMG. Mechanism analysis uncovers that the magnetic multiplier facilitates the regulation of transmission ratio and assists in achieving a faster operating frequency of EMG than TENG. The parameters affecting RTENG (grating degree of electrode, frequency, and tribo-layer) and EMG (transmission ratio and frequency) outputs are systematically studied. The proposed strategy allows for an effective energy utilization efficiency of EMG comparable to that of RTENG at the same driving frequency, which is a substantial improvement in comparison to the hybrid generator's operation at cofrequency. Moreover, the magnetic-multiplier-enabled hybrid generator (MMHG) can be used to build self-powered water quality monitoring and fishing alarm systems benefiting from a power management circuit (PMC). This work renders a feasible approach toward low-frequency rotational energy harvesting with high energy utilization efficiency by means of division frequency strategy.

## Results

### Device structure and working mechanism

**Fig. 1.**
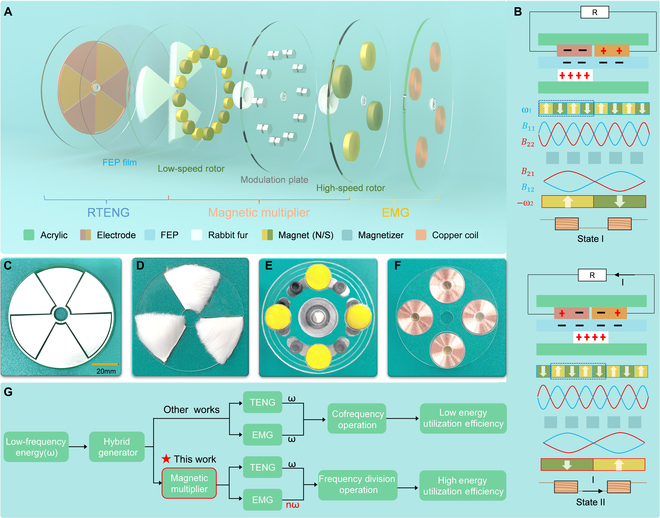
Structure and working mechanism of the MMHG. (A) Exploded view of the MMHG, including a RTENG, a magnetic multiplier, and a coil panel. (B) Working mechanism of MMHG. (C to F) Digital photographs of the 60° grating electrode, rabbit fur, magnetic multiplier with 1:4 transmission ratio, and copper coils, which works together to construct the MMHG. (G) Comparison of TENG and EMG energy utilization efficiency in hybrid generators with different operating modes.

An MMHG is proposed to address the challenge of achieving optimal energy utilization efficiency of both TENG and EMG when they operate at the same frequency within a hybrid generator. The MMHG has a layer structure including an RTENG, a magnetic multiplier, and a coil panel. The exploded view in Fig. 1A shows that the fluorinated ethylene propylene (FEP) film over the grating electrodes and the soft rabbit fur are employed as the tribo-materials. The magnetic multiplier is positioned in the middle and composed of a low-speed rotor, a modulation plate, and a high-speed rotor, where the 2 rotors are made up of round magnets with alternating south and north poles. The transmission ratio can be controlled by modifying the number of magnets and magnetizers. Additionally, the high-speed rotor serves to form an EMG with the coil panel. Given the alternating arrangement of the magnets in north–south directions, the adoption of the 4-coil interconnection approach, as exemplified in Fig. S1, constitutes a method to attain the highest degree of magnetic flux variation. Two side views in Fig. 1B illustrate the working mechanism of MMHG. Initially, when an external force is applied to TENG, tribo-charges are created at the interface between the rabbit fur and FEP according to the triboelectrification effect. The cyclic departure and approach to the rabbit fur sector result in free charges to redistribute between 2 electrodes behind the FEP to balance the potential difference, generating an alternating current signal in the external circuit. The operating frequency of TENG remains consistent with the driving frequency (*ω*). As for EMG, changes in the magnetic field during rotation create electrical signals in the coil, which depends on the rotating frequency of the high-speed rotor (*nω*) instead of the low-speed rotor (driving frequency). Taking the magnetic multiplier with a transmission ratio of 1:4 as an example, a fundamental wave *B*_11_ (blue line) generated by the low-speed rotor with 4 pairs of magnetic poles forms a harmonic wave *B*_12_ (blue line) by the fixed modulation plate with 5 magnetizers. The airgap flux density created by the permanent magnet in the low-speed rotor can be expressed as:

$$B_{11}=\Lambda_{0}F_{1m}\cos\left[p_{1}\left(\theta -c_{1}\omega_{1}t\right)+\varphi_{1}\right]$$ (1)

$$B_{12}=\frac{1}{2}\Lambda_{m}F_{1m}\cos\left[\left(N-p_{1}\right)\left(\theta -\frac{\mathrm{cN}\omega -c_{1}p_{1}\omega_{1}}{N-p_{1}}t\right)+\left(\varphi -\varphi_{1}\right)\right]$$ (2)

where *Λ*_0_ is the mean component of the magnetic conductance and *F*_1m_ and *φ*_1_ are the magnitudes and initial phase angle of magnetomotive force in low-speed rotor, respectively. *θ* represents the mechanical angle. *c*_1_ is the steering coefficient of the low-speed rotor, with a value of 1 for clockwise rotation and −1 for counterclockwise rotation. Similarly, the high-speed rotor with 2 pairs of magnetic poles also produces a fundamental wave *B*_21_ (red line) and harmonic wave *B*_22_ (red line). The airgap flux density generated by the permanent magnet in the high-speed rotor can be described as:

$$B_{21}=\Lambda_{0}F_{2m}\cos\left[p_{2}\left(\theta -c_{2}\omega_{2}t\right)+\varphi_{2}\right]$$ (3)

$$B_{22}=\frac{1}{2}\Lambda_{m}F_{2m}\cos\left[\left(N-p_{2}\right)\left(\theta -\frac{\mathrm{cN\omega}-c_{2}p_{2}\omega_{2}}{N-p_{2}}t\right)+\left(\varphi -\varphi_{2}\right)\right]$$ (4)

where *F*_2m_ and *φ*_2_ are the magnitudes and initial phase angle of the magnetomotive force in the high-speed rotor, respectively. *c*_2_ is the steering coefficient of the high-speed rotor. The coupling between *B*_11_ and *B*_22_, *B*_21_ and *B*_12_ produces 2 stable magnetic torques, facilitating the high-speed rotor follow the movement of low-speed rotor without any physical contact. In addition, the high-speed rotor rotates in the opposite direction to the low-speed rotor, with an angular frequency of *ω*_2_ when the latter rotates at a frequency of *ω*_1_. The detailed working principle of the magnetic multiplier is displayed in Note S1. The relationship between *ω*_1_ and *ω*_2_ is the transmission ratio *i*, given by

$$i=\frac{\omega_{2}}{\omega_{1}}=\frac{p_{1}}{p_{2}}$$ (5)

where *p*_1_ and *p*_2_ are the number of pole pairs on the low-speed rotor and the high-speed rotor, respectively, equal to half the number of magnets actually used. What is more, the number of the magnetizers *N* is obtained by

$$N=p_{1}+p_{2}$$ (6)

Figure 1C is the photograph of grating electrode and the tribo-layer (rabbit fur) matched the electrode pattern is shown in Fig. 1D. The well-prepared magnetic multiplier with a transmission ratio of 1:4 and the coil panel are displayed in Fig. 1E and F. To provide a comprehensive view, Fig. S2 exhibits the magnetic multipliers with transmission ratios of 1:2 and 1:3, as well as the 45°, 60° grating electrode, along with their respective tribo-layer. Since the introduction of the first HETG in 2015, most of the devices developed so far operate in the cofrequency mode, leading to low energy utilization efficiency due to the mismatch between the optimal operating frequencies of TENG and EMG. As such, we propose a strategy to improve energy utilization efficiency by implementing a frequency division approach that enables EMG to operate at high frequencies through a magnetic multiplier, while TENG works at low frequencies to avoid sacrificing its output as shown in Fig. 1G.

### Electrical characteristics of RTENG

Before evaluating the performance of MMHG, a series of experiments are conducted to measure the electrical output of RTENG. The corresponding test platform is established in Fig. 2A, where the MMHG is arranged vertically on a sliding rail and driven by a motor. To optimize the optimal structural parameters of RTENG, the effects of electrode grating degree, frequency, and tribo-layer on device’s output performance are systematically investigated. The transferred charge curves of 30°, 45°, and 60° grating electrodes cooperating with the rabbit fur at 2.67 Hz are plotted in Fig. 2B, indicating that the magnitude of transferred charge increases while wave number obtained in 1 cycle decreases with the grating degree of electrode varying from 30° to 60°. A general view of the 3 types of electrodes is also displayed in Fig. 2B. As plotted in Fig. 2C, both open-circuit voltage (*V*_oc_) and transferred charge of RTENG with 3 grating electrodes initially show a growth trend with the frequency going from 0.67 to 2.67 Hz and then trend to saturation as the frequency keeps increasing. That is, the surface charges generated on the tribo-layers approach saturation at 2.67 Hz. Hence, this frequency is selected for the follow-up experiments. The waveforms for each data point in Fig. 2C are summarized in Fig. S3. Notably, the *V*_oc_ of RTENG exceeds the measurement range of Keithley 6514, so it is studied by the voltage division method. Moreover, because the internal resistance of RTENG is on the order of megohm, the external resistance should be sufficiently large to ignore the internal resistance. Thus, 2 resistors (*R*_1_ = 600 GΩ,  *R*_2_ = 1 GΩ) are connected in series to both ends of the RTENG to concentrate all the voltage on the 2 resistors. The circuit diagram is depicted in Fig. S4. Based on this, the *V*_oc_ of RTENG is calculated by

$$V_{\mathrm{oc}}=\frac{R_{1}+R_{2}}{R_{2}}V$$

**Figure:**
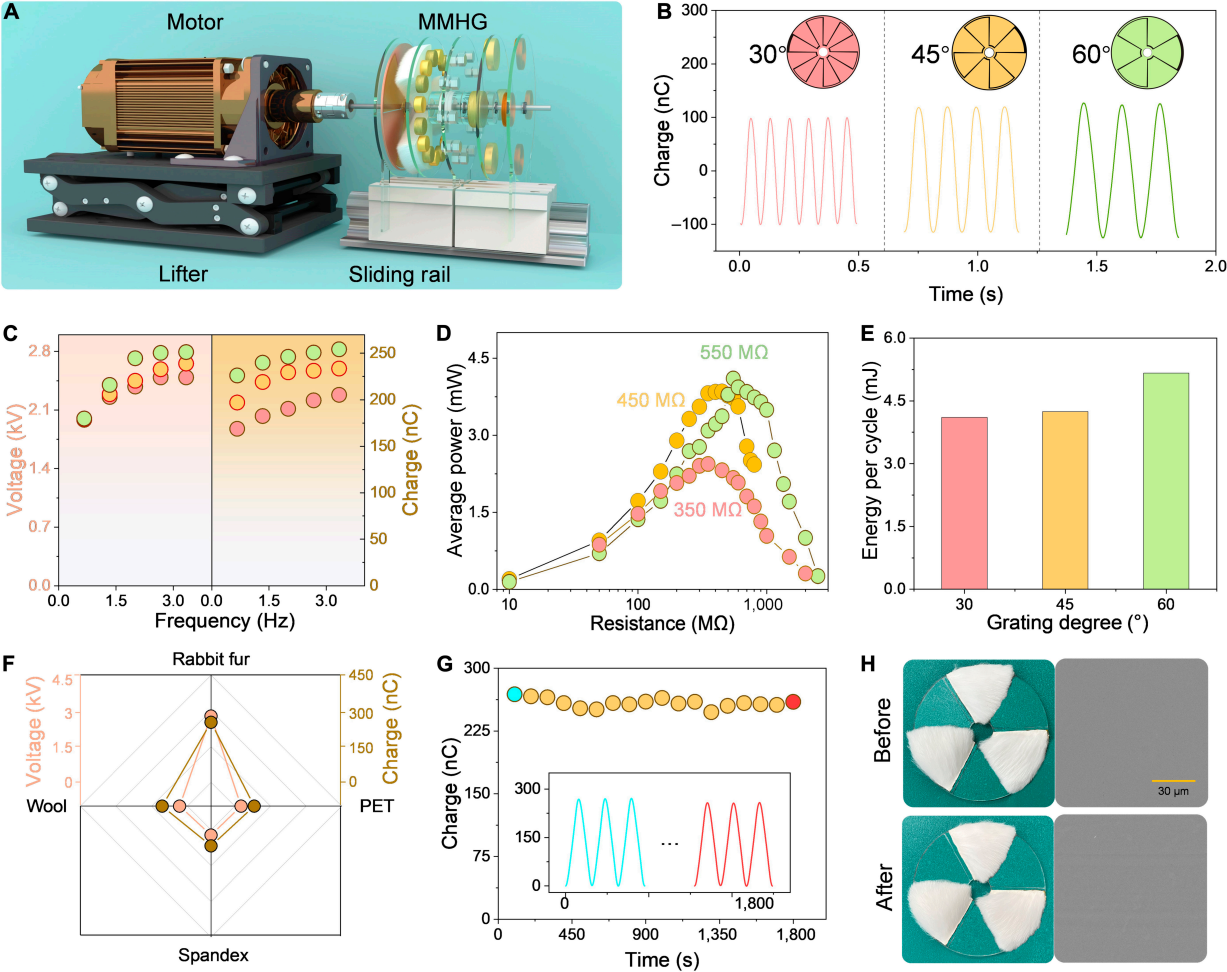
Electrical characteristics of RTENG. (A) The test platform consisting of a sliding rail, a lifter, a motor, and the MMHG. (B) Transferred charge curves of 30°, 45°, and 60° grating electrodes cooperating with the rabbit fur, obtained in 1 cycle at 2.67 Hz. Insets are the general view of 3 kinds of electrodes. (C) Dependence of open-circuit voltage and transferred charge of 3 different electrodes on frequency. (D) Average power of 30°, 45°, and 60° grating electrodes under external load resistances at 2.67 Hz. (E) Comparison of the output energy for 3 electrodes in 1 cycle at the matched impedance (2.67 Hz). (F) Transferred charge and open-circuit voltage of 4 kinds of tribo-layers (rabbit fur, wool, spandex, and PET). (G) Durability test of RTENG with FEP film and rabbit fur. Insets are the charge waveform at the beginning (blue line) and the end (red line) of the test. (H) Photographs of the rabbit fur and SEM images of the FEP film before and after the durability test. The device is driven by a motor.

where *V* is the voltage across *R*_2_. The average output power at different external load resistances is presented in Fig. 2D, which is expressed as

$$\bar{P}=\frac{\int_{0}^{T}I^{2}\mathrm{dt}}{T}R$$ (8)

where *R* is the load resistance and *I* is the instantaneous current across the resistance. The results manifest that the average power of 30°, 45°, and 60° grating electrodes is delivered to its maximum value of 2.442, 3.850, and 4.107 mW at the matched resistance of 350, 450, and 550 MΩ, respectively. Notably, with an increase in the grating angle of electrode, a corresponding rise in the matched resistance of the RTENG ensues. By considering the RTENG as a capacitor and effectuating its connection with an external resistor, an RC oscillation circuit is engendered. The relationship between frequency (*f*) and capacitance (*C*) and resistance (*R*) is denoted by

$$f=\frac{1}{2\pi \mathrm{RC}}$$ (9)

Due to the capacitance (*C*) maintains constant across these configurations, an augmentation in the grating electrode angle yields a reduction in the operational frequency. Consequently, the matching resistance of the RTENG, as exhibited by the 30°, 45°, and 60° grating electrodes, gradually experiences an incremental ascent. At the matched resistance, the energy accumulated in 1 cycle is indicated as

$$W=\int_{0}^{T}I^{2}R_{m}\mathrm{dt}$$ (10)

where *T* is the time to perform 1 rotation and *R_m_* is the matched resistance. The integral graphs of *I*^2^ are described in Fig. S5. The accumulated energies of 30°, 45°, and 60° grating electrodes in 1 cycle are 4.108, 4.246, and 5.161 mJ, respectively (Fig. 2E).

Ultimately, the 60° grating electrodes is chosen for the subsequent measurements based on its superior output performance. In terms of the tribo-layer, fur products are evaluated as the positive tribo-layer with a slight wear. Two pairs of natural fibers (rabbit fur, wool) and 2 types of artificial fibers (spandex and polyethylene terephthalate [PET]) were tested. The results show that the rabbit fur produces the best electric signals among all 4 types of tribo-layers, evidenced in the associated waveform in Fig. 2F. The voltages generated by the other 3 materials fall within a measurable range and are directly measured by Keithley 6514. Figures S6 and S7 are the photographic depictions of the other 3 materials and their respective waveforms. After 1,800 s of continuous durability testing, no noticeable change is observed in the output performance of RTENG, except for the rabbit fur becoming disheveled and minor scratches appear on the surface of FEP film (Fig. 2G and H), indicating that the RTENG has good stability and durability.

### Electrical characteristics of EMG part

**Fig. 3.**
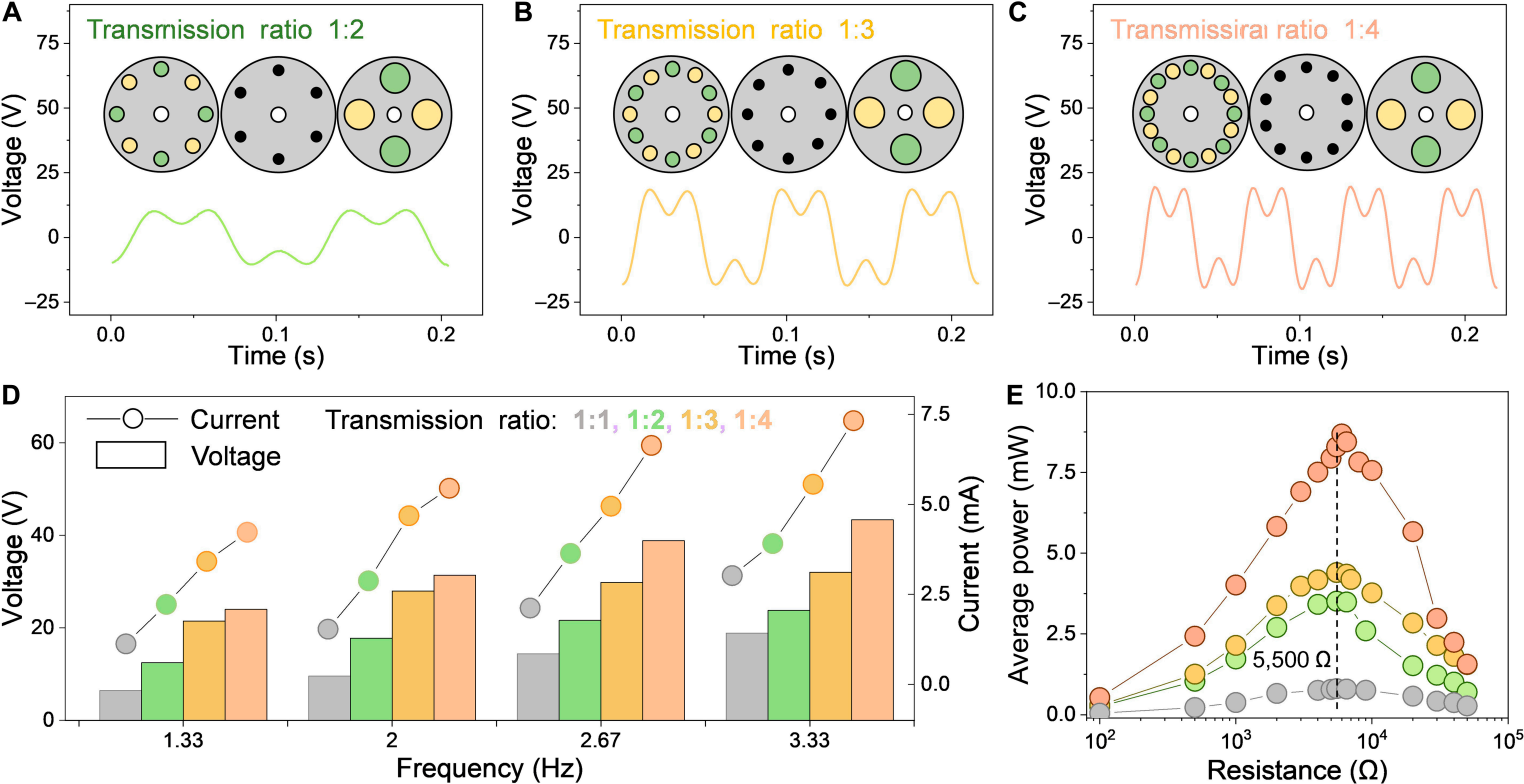
Electrical characteristics of magnetic multiplier enabled EMG. (A to C) Open-circuit voltage curves of EMG with 3 transmission ratios magnetic multipliers (1:2, 1:3, and 1:4). Insets are the schematic diagrams of low-speed rotor, modulation plate, and high-speed rotor for 3 magnetic multipliers. (D) Dependence of open-circuit voltage and short-circuit current on frequency. (E) Average power of EMG with 4 transmission ratios under external load resistances. The device is driven by a motor.

To enhance the energy utilization efficiency of EMG at low driving frequency, a frequency division approach is constructed by a magnetic multiplier. In that case, TENG operates at the same frequency as the drive source, while EMG operates at a higher frequency determined by the transmission ratio of the magnetic multiplier. Three devices with transmission ratios of 1:2, 1:3, and 1:4 are designed within the size limitation of MMHG and magnets. Schematic plots of the low-speed rotor, modulation plate, and high-speed rotor for 3 magnetic multipliers are depicted in Fig. 3A to C, among which yellow and green circles represent the 2 poles of magnets and black circles denote the magnetizers. The number of magnets and magnetizers depends on the above Eqs. 5 and 6. The magnetic field generated by the low-speed and high-speed rotors in the magnetic multiplier forms a stable coupling harmonic pair through the modulation plate. The resulting interaction of the coupling harmonic pairs causes a reverse speed-up transmission between the low-speed and high-speed rotors. The working process, as recorded in Movie S1, indicates that when the motor-driven low-speed rotor completes 1 turn, the high-speed rotor rotates reversely through the corresponding turns as per the transmission ratio. This confirms that the designed magnetic multipliers functioned as expected. The low-speed rotor shares an acrylic substrate with the rabbit fur of RTENG and can be driven directly by external forces, transmitting motion to the high-speed rotor via the magnetic torque and inducing an ac signal in the coils to achieve frequency division operation. If the hybrid generator is designed to work at the same frequency by coupling the coil panel with the low-speed rotor, the magnetic field's change rate can be kept constant by increasing the number of coils. From the theoretical calculation demonstrated in Note S2, the output of EMG will decrease at a fixed device volume, underscoring the necessity of using a high-speed rotor to construct the EMG.

Referring to Fig. 3A to C and Fig. S8, when the transmission ratio varies from 1:1 to 1:4, the peak-to-peak voltage of EMG increases from 13.962 to 38.936 V. The depression between 2 peaks is caused by the magnet’s distribution on the high-speed rotor, which results in 2 voltage waveforms that do not overlap at the peaks. Additionally, it can be observed in Fig.3D that both open-circuit voltage and short-circuit current of the 4 EMGs show a conspicuous growth trend with increasing the frequency from 1.33 to 3.33 Hz at any transmission ratio, in line with Faraday’s principle of electromagnetic induction. Similarly, when the transmission ratio changes from 1:1 (purple) to 1:4 (red), the operating frequency of EMG is increased by 4 times at the same driving frequency, which greatly improves the output performance of EMG. The current of EMG at various external load resistances is characterized at 2.67 Hz. According to the Eq. 8, the calculated average power shows that at the matched resistance of 5,500 Ω, the maximum powers delivered by 4 EMGs (1:1, 1:2, 1:3, and 1:4) reach 0.811, 3.515, 4.401, and 8.688 mW, respectively (Fig. 3E).

### Output performance of the power-managed MMHG

**Fig. 4.**
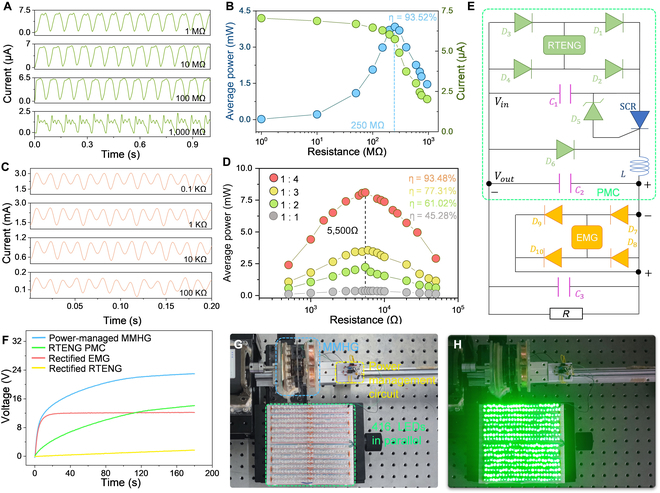
Output performance of the power-managed MMHG. (A) Current curves of rectified RTENG supplied to different external resistances (1, 10, 100, and 1,000 MΩ). (B) Average power and peak current of RTENG after rectification under external load resistances. (C) Current curves of rectified EMG with a 1:4 transmission ratio magnetic multiplier supplied to different external resistances (0.1, 1, 10, and 100 KΩ). (D) Average power of the EMG of 4 transmission ratios after rectification at external load resistance. (E) Schematic of the circuit for power-managed MMHG. (F) Charging performances of a 470-μF capacitator by the direct rectified RTENG, RTENG with PMC, direct rectified EMG, and power-managed MMHG. (G) Photograph of the powered-managed MMHG as a power source to light 416 light-emitting diodes (LEDs) in parallel connection. (H) Photograph of the lighted state. The device is driven by a motor at 2.67 Hz.

Based on the above results, a hybrid generator with optimized parameters is fabricated. Both EMG and RTENG give alternating signals, but only direct current is required for electronics, which mandates the use of rectifiers in EMG and RTENG. Unfortunately, energy loss at the rectification circuit is inevitable and the energy utilization efficiency, which describes the degree of energy loss, is calculated by

$$\eta =\frac{P_{a}}{P_{b}}$$ (11)

where *P_b_* and *P_a_* indicate the maximum average power before and after direct rectification, respectively. The waveforms of the rectified current in the RTENG, with different external load resistances, are demonstrated in Fig. 4A. As the external resistance increases from 1 to 1,000 MΩ, the peak current value decreases, and more extensive data are presented in Fig. 4B. The maximized average output power is 3.84 mW at the matched resistance of 250 MΩ, and the integral curves of *I*^2^ are available in Fig. S9. Combining with the maximized average output power before rectification (Fig. 2D), the energy utilization efficiency of RTENG reaches 93.52% at 2.67 Hz. Similarly, the rectified current of EMG decreases as the external load resistances varies from 0.1 to 100 kΩ, exhibited in Fig. 4C. The maximum average power of the 4 EMGs after rectification (1:1, 1:2, 1:3, and 1:4) is 0.37, 2.23, 3.56, and 8.12 mW (Fig. 4D). Compared to the conclusions in Fig. 3E, the energy utilization efficiency reaches 45.28%, 61.02%, 77.31%, and 93.48%, respectively, which demonstrates a obvious improvement in the utilization efficiency by introducing the magnetic multipliers to achieve the frequency division strategy.

In order to match the charging ability of EMG and RTENG, a PMC that contains a full-bridge rectifier (D_1_-D_4_), a silicon-controlled rectifier (SCR), and a Zener diode (D_5_), 2 capacitors (C_1_ and C_2_), and an inductor (L) is used to endow RTENG a rapid charge capacity for large capacitances, as the part enclosed by a dotted green wireframe in Fig. 4E. Initially, the energy generated by RTENG is stored in C_1_ via the full-bridge rectifier and the voltage of C_1_ quickly rises toward the maximal value. When the voltage across C1 exceeds the breakdown voltage of D_5_ + VC_2_, D_5_ becomes reverse conduction, resulting in an injection of current into the SCR gate and triggering its conductance. Thus, the voltage on SCR drops to 0 instantaneously. When VC_1_ decreases to nearly 0, D_6_ begins to forward conduct that locks the voltage of C_1_ around 0 and keeps the current through C_1_ and SCR at 0. In this case, the SCR is equivalent to entering the off state and waiting for the next current injection. Eventually, the overall output of MMHG is obtained by serial connection of the rectified EMG and RTENG with PMC (the connection mode is given in Fig. 4E). With the help of PMC, the charging rate for a 470 μF capacitor reaches 12 V/min, whereas the direct rectified RTENG only produces 0.58 V/min charging rate. Due to the high output current but low output voltage, the direct rectified EMG can elevate the voltage of capacitor to a saturation state of 12 V in 23 s. The low saturated voltage but fast charging process of the EMG provides a platform on which the voltage of the capacitor can be further improved by TENG, as depicted in Fig. 4F (blue line). Working as a whole, the powered-managed MMHG is able to generate electricity from the motor rotation to illuminate 416 green light-emitting diodes in parallel connection, as shown in Fig. 4G and H.

### Application of the power-managed MMHG

**Fig. 5.**
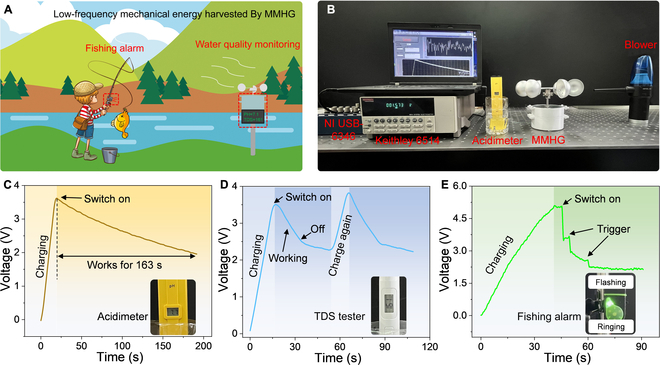
Application of the power-managed MMHG. (A) Typical application scenario of the power-managed MMHG in water quality monitoring and fishing alarm. (B) Experimental platform for simulating the scenario of harvesting wind energy to power the acidimeter. (C) Charging and discharging process of a 9.4-mF capacitor in powering the acidimeter by airflow-driven MMHG, indicating the acidimeter works 163 s after charging in 20 s. (D) Working waveform of the 9.4-mF capacitor in powering the water quality tester by airflow-driven MMHG. (E) Voltage curve of charging an 18.8-mF capacitor to power a fishing alarm by hand-driven MMHG. The respective working status is recorded in the inset.

To explore the capability of the power-managed MMHG in harnessing low-frequency energy as a practical power source, a typical application scenario is presented and illustrated in Fig. 5A. The power-managed MMHG is applied to harvest the wind energy to monitor local water quality in both PH and total dissolved solids (TDS) with the aid of a wind cup, as well as being integrated into the rod rocker to establish a fishing alarm. Figure 5B is a photograph of the experimental platform used to simulated wind energy collection. A 9.4-mF capacitor is charged by the power-managed MMHG at a wind of 8.6 m/s. The capacitor’s voltage reaches 3.62 V in 20 s, which allows the acidimeter to operate for 163 s without the need for recharging. The voltage curve and working process are exhibited in Fig. 5C and Movie S2. Besides, TDS, another indicator of water quality, is also surveyed. The 9.4-mF capacitor is first charged to 3.51 V by MMHG in 16 s and then connected to the water quality tester, which keeps the tester working 28 s until the voltage drops to 2.3 V. When the capacitor is recharged to 3.76 V, the tester works for 45 s (Fig. 5D and Movie S3). Figure 5E shows the charging curve of an 18.8-mF capacitor for powering a fishing alarm. The hands rotational energy scavenged by MMHG is first stored in capacitors and then serviced to supply energy for the alarm. When the fishing line on the movable rod is pulled by a fish, the small bells emit sound and flash to remind the fisherman that the fish is hooked. Two triggers are maintained at a single charge. The detailed work process is recorded in Movie S4. All applications demonstrate the superior capabilities of the MMHG in low-frequency energy harvesting.

## Discussion

In this work, we designed a MMHG to facilitate the 2 parts of MMHG to function simultaneously at divided frequency. TENG moves at the same frequency as the low-speed rotor of the magnetic multiplier, while the output of EMG relies on the working frequency of the high-speed rotor, which is achieved through internal harmonic magnetic field coupling. Based on this structure design, systematically investigations about the factors affecting both RTENG and EMG outputs are implemented. Under the action of the rectification bridge, the effective energy utilization efficiency of EMG reaches the same level of RTENG. Furthermore, incorporating with a PMC, the MMHG can continuously supply power for acidimeter, TDS tester, and fish alarm, establishing a self-powered water quality and fishing monitoring system. This frequency division strategy demonstrated in this work provides an effective way for hybridized generators to achieve high energy utilization efficiency in low-frequency rotational energy harvesting, which paves stepping stones for further development of the self-powered systems.

## Materials and Methods

### Fabrication of MMHG

The MMHG is mainly divided into 3 sections, concluding a RTENG, a magnetic multiplier, and a coil panel. For the RTENG part, a FEP film with 70-μm thickness is covered on the grating electrode (diameter: 82 mm) as a tribo-layer. Three sets of complementary grating-arrayed electrodes are patterned by the print circuit board technology (30°, 45°, and 60°). Another tribo-layer is the rabbit fur shaped to the half pattern of grating electrode, which is stuck to an acrylic sheet (thickness: 2 mm, diameter: 85 mm). As to the magnetic multiplier portion, a set number of small magnets (thickness: 3.75 mm, diameter: 10 mm) are placed alternating north and south around the center of a circular acrylic with 2-mm thickness and 85-mm diameter, which constitutes the low-speed rotor. And a certain number of screws and nuts (size: M5) are fixed in a circle to the acrylic sheet (thickness: 2 mm, diameter: 85 mm) to form the modulation plate. Same as the low-speed rotor, the high-speed rotor is made up of 4 big magnets (thickness: 3.65 mm, diameter: 20 mm). Two flat thrust ball bearings are used to separate the 3 disks and reduce friction between them. The EMG unit is built by the high-speed rotor of magnetic multiplier and the coil panel that includes 4 coils. The wire diameter and the resistance of each coil is 0.08 mm and 1.5 kΩ. Finally, a stainless-steel solid round rod (length: 100 mm, diameter: 7 mm) is used as the central axis to connect the 3 segments. In addition, 4 bearing (outer diameter: 11 mm, inner diameter: 7 mm) are installed in the electrode of RTENG, the modulation plate and high-speed rotor of magnetic multiplier, and the coil panel to ensure that these 4 positions do not rotate with external forces.

### Electric measurements

A motor (3RK15RGN-C) is adopted to provide steady and adjustable rotational speed. A programmable electrometer (Keithley 6514) is applied to investigate the output voltage, transferred charge, and output current of device. The date is collected by National Instruments acquisition card (National Instruments USB-6346), and the LabView program is utilized to control and monitor real-time date acquisition.

## Data Availability

We declare that the main data supporting the findings of this study are available within the article and Supplementary Materials. Extra data are available from the corresponding authors on reasonable request.

## Supplementary Materials

Supplementary 1 Note S1. The basic working principle of the magnetic multiplier. Note S2. The performance of EMG made by the high-speed and low-speed rotors. Fig. S1. Schematic diagram of coil’s connection in EMG. Fig. S2. (A and B) Photographs of 45°, 30° grating electrode. (C and D) Photographs of 45° and 30° rabbit fur tribo-layer. (E and F) Photographs of magnetic multiplier with transmission ratios of 1:2 and 1:3. Fig. S3. Transferred charge and open-circuit voltage curves of 30°, 45°, and 60° grating electrode obtained in 1 cycle at different frequencies (0.67, 1.33, 2, 2.67, and 3.33 Hz). Fig. S4. Circuit diagram of the voltage division method. Fig. S5. Integral curve of 30°, 45°, and 60° grating electrode obtained in 1 cycle. Fig. S6. Photographs of wool, spandex, and PET corresponding to 60° grating electrode. Fig. S7. (A to C) Open-circuit voltage and transferred charge curves of the wool, spandex, PET-based TENG. Fig. S8. Open-circuit voltage curves of EMG without magnetic multiplier (transmission ratio 1:1). Fig. S9. Integral curve of series external resistors (1, 10, 50, 100, 150, 200, 250, 300, 400, 600, 700, and 750 MΩ).

Supplementary 2 Movie S1. Display the magnetic multiplier. Movie S2. MMHG powers the acidimeter. Movie S3. MMHG powers the water quality tester. Movie S4. MMHG powers the fishing alarm.
