# Supplementary material for: A Magnetic-Multiplier-Enabled Hybrid Generator with Frequency Division Operation and High Energy Utilization Efficiency
Source: Research (Wash D C). 2023 Jun 9;6:0168. doi: 10.34133/research.0168 (PMC10254463; doi:10.34133/research.0168)
Supplement: Supplementary 1 — Note S1. The basic working principle of the magnetic multiplier. Note S2. The performance of EMG made by the high-speed and low-speed rotors. Fig. S1. Schematic diagram of coil’s connection in EMG. Fig. S2. (A and B) Photographs of 45°, 30° grating electrode. (C and D) Photographs of 45° and 30° rabbit fur tribo-layer. (E and F) Photographs of magnetic multiplier with transmission ratios of 1:2 and 1:3. Fig. S3. Transferred charge and open-circuit voltage curves of 30°, 45°, and 60° grating electrode obtained in 1 cycle at different frequencies (0.67, 1.33, 2, 2.67, and 3.33 Hz). Fig. S4. Circuit diagram of the voltage division method. Fig. S5. Integral curve of 30°, 45°, and 60° grating electrode obtained in 1 cycle. Fig. S6. Photographs of wool, spandex, and PET corresponding to 60° grating electrode. Fig. S7. (A to C) Open-circuit voltage and transferred charge curves of the wool, spandex, PET-based TENG. Fig. S8. Open-circuit voltage curves of EMG without magnetic multiplier (transmission ratio 1:1). Fig. S9. Integral curve of series external resistors (1, 10, 50, 100, 150, 200, 250, 300, 400, 600, 700, and 750 MΩ). [file research.0168.f1.docx]

Supplementary Materials

**Supplementary Materials including:**

Note S1. The basic working principle of the magnetic multiplier.

Note S2. The performance of EMG made by the high speed and low speed rotors.

Fig. S1. Schematic diagram of coil’s connection in EMG.

Fig. S2. (a)-(b) Photographs of 45°, 30° grating electrode. (c)-(d) Photographs of 45°, 30° rabbit fur tribo-layer. (e)-(f) Photographs of magnetic multiplier with transmission ratios of 1:2 and 1:3.

Fig. S3. Transferred charge and open-circuit voltage curves of 30°, 45°, 60° grating electrode obtained in one cycle at different frequencies (0.67 Hz, 1.33 Hz, 2 Hz, 2.67 Hz, 3.33 Hz).

Fig. S4. Circuit diagram of the voltage division method.

Fig. S5. Integral curve of 30°, 45°, 60° grating electrode obtained in one cycle.

Fig S6. Photographs of Wool, Spandex, PET corresponding to 60° grating electrode.

Fig. S7. (a)-(c) Open-circuit voltage and transferred charge curves of the Wool, Spandex, PET based TENG.

Fig. S8. Open-circuit voltage curves of EMG without magnetic multiplier (transmission ratio 1:1).

Fig.9. Integral curve of series external resistors (1, 10, 50, 100, 150, 200, 250, 300, 400, 600, 700, 750 MΩ).

**Other Supplementary Materials for this work includes the following:**

Movie S1. Display the magnetic multiplier.

Movie S2. MMHG powers the acidimeter.

Movie S3. MMHG powers the water quality tester.

Movie S4. MMHG powers the fishing alarm.

**Supplementary Note 1**

To describe the operation of a magnetic multiplier, the angular velocity of low-speed rotor and high-speed rotor are defined as $\omega_{1}$ and $\omega_{2}$, respectively, while the number of pole pairs are $p_{1}$ and $p_{2}$. Additionally, the angular velocity of modulation plate is $\omega$ and the number of the magnetizers is expressed as N. Assuming that the magnetomotive forces of the permanent magnets on the low-speed rotor and high-speed rotor are distributed in a sinusoidal pattern along the circumference.

$F_{1}=F_{1m}cos[p_{1}\left( \theta-c_{1}\omega_{1}t \right)+\varphi_{1}$ (S1)

$F_{2}=F_{2m}cos[p_{2}\left( \theta-c_{1}\omega_{2}t \right)+\varphi_{2}]$ (S2)

where $F_{1m}$ and $F_{2m}$ are the magnitudes of magnetomotive force in low-speed rotor and high-speed rotor, $\varphi_{1}$ and $\varphi_{2}$ are the initial phase angle of the magnetomotive force in the low-speed rotor and high-speed rotor, $\theta$ is the mechanical angle. $c_{1}$ and $c_{2}$ are the steering coefficient of the low-speed rotor and high-speed rotor, with a value of 1 for clockwise rotation and -1 for counterclockwise rotation. The magnetic conductance of the modulation plate can be expressed as

$\Lambda=\Lambda_{0}+\Lambda_{m}cos[N\left( \theta-c\omega t \right)+\varphi$ (S3)

where $\Lambda_{0}$ is the mean component of the magnetic conductance, $\Lambda_{m}$ is the amplitude of the alternating component of the magnetic conductance,$\varphi$ is the initial phase angle of the alternating component of the magnetic conductance, and c is the steering coefficient of the magnetic conductance. A value of -1 indicates counterclockwise rotation and a value of 1 indicates clockwise rotation. The airgap flux density generated by the permanent magnet in the low-speed rotor can be obtained from Eq. S1 and Eq. S3:

$B_{1}=\Lambda F_{1}=\Lambda_{0}F_{1m}\cos\left[ p_{1}\left( \theta-c_{1}\omega_{1}t \right)+\varphi_{1} \right]+\frac{1}{2}\Lambda_{m}F_{1m}\cos\left[ \left( N-p_{1} \right)\left( \theta-\frac{cN\omega-c_{1}p_{1}\omega_{1}}{N-p_{1}}t \right)+\left( \varphi-\varphi_{1} \right) \right]+\frac{1}{2}\Lambda_{m}F_{1m}\cos\left[ \left( N+p_{1} \right)\left( \theta-\frac{cN\omega+c_{1}p_{1}\omega_{1}}{N+p_{1}}t \right)+\left( \varphi+\varphi_{1} \right) \right]=B_{11}+B_{12}+B_{13}$ (S4)

$B_{11}=\Lambda_{0}F_{1m}\cos\left[ p_{1}\left( \theta-c_{1}\omega_{1}t \right)+\varphi_{1} \right]$ (S5)

$B_{12}=\frac{1}{2}\Lambda_{m}F_{1m}\cos\left[ \left( N-p_{1} \right)\left( \theta-\frac{cN\omega-c_{1}p_{1}\omega_{1}}{N-p_{1}}t \right)+\left( \varphi-\varphi_{1} \right) \right]$ (S6)

$B_{13}=\frac{1}{2}\Lambda_{m}F_{1m}\cos\left[ \left( N+p_{1} \right)\left( \theta-\frac{cN\omega+c_{1}p_{1}\omega_{1}}{N+p_{1}}t \right)+\left( \varphi+\varphi_{1} \right) \right]$ (S7)

Similarly, the airgap flux density generated by the permanent magnet in the high-speed rotor can be obtained from Eq. S2 and Eq. S3:

$B_{2}=B_{21}+B_{22}+B_{23}$ (S8)

$B_{21}=\Lambda_{0}F_{2m}\cos\left[ p_{2}\left( \theta-c_{2}\omega_{2}t \right)+\varphi_{2} \right]$ (S9)

$B_{22}=\frac{1}{2}\Lambda_{m}F_{2m}\cos\left[ \left( N-p_{2} \right)\left( \theta-\frac{cN\omega-c_{2}p_{2}\omega_{2}}{N-p_{2}}t \right)+\left( \varphi-\varphi_{2} \right) \right]$ (S10)

$B_{23}=\frac{1}{2}\Lambda_{m}F_{2m}\cos\left[ \left( N+p_{2} \right)\left( \theta-\frac{cN\omega+c_{2}p_{2}\omega_{2}}{N+p_{2}}t \right)+\left( \varphi+\varphi_{2} \right) \right]$ (S11)

It can be observed that the airgap magnetic field generated by the permanent magnets in the low-speed and high-speed rotor comprises three components. The first component ($B_{11}$ and $B_{21}$) is fundamental component, whose number of pole-pairs and rotate speed are equal to their respective rotor (low-speed rotor and high-speed rotor). The second ($B_{12}$ and $B_{22}$) and third ($B_{13}$ and $B_{23}$) components are generated by the modulation effect of the modulation plate. If the rotate speed of the modulation plate and the initial phase angle of the modulation plate and low-speed rotor are 0, Eq. S6 and Eq. S7 can be simplified to:

$B_{12}=\frac{1}{2}\Lambda_{m}F_{1m}\cos\left[ \left( N-p_{1} \right)\left( \theta-\frac{-c_{1}p_{1}\omega_{1}}{N-p_{1}}t \right) \right]$ (S12)

$B_{13}=\frac{1}{2}\Lambda_{m}F_{1m}\cos\left[ \left( N+p_{1} \right)\left( \theta-\frac{c_{1}p_{1}\omega_{1}}{N+p_{1}}t \right) \right]$ (S13)

Therefore, the number of pole-pairs on $B_{12}$ is $N-p_{1}$ and the rotate speed is $\frac{p_{1}}{N-p_{1}}$ which has the opposite direction with $B_{11}$. If the number of magnetizers satisfies

$N=p_{1}+p_{2}$ (S14)

then Eq. S12 is transformed into

$B_{12}=\frac{1}{2}\Lambda_{m}F_{1m}\cos\left[ p_{2}\left( \theta-\frac{-c_{1}p_{1}\omega_{1}}{N-p_{1}}t \right) \right]$ (S15)

In this manner, the number of pole-pairs on $B_{12}$ is $p_{2}$, which is equivalent to the first component $B_{21}$. This creates stable coupling between $B_{12}$ and $B_{21}$ (the number of pole-pairs on $B_{13}$ is not equal to $B_{21}$, so $B_{13}$ and $B_{21}$ will not couple with each other). Homoplastically, stable coupling is also formed between $B_{11}$ and $B_{22}$ (the number of pole-pairs on $B_{23}$ is not equal to $B_{11}$, so $B_{23}$ and $B_{11}$ will not couple with each other). The relationship between $\omega_{2}$ and $\omega_{1}$is given by the transmission ratio $i$, which can be expressed as

$i=\frac{\omega_{2}}{\omega_{1}}=\frac{p_{1}}{p_{2}}$ (S16)

That is the basic working principle of the magnetic multiplier.

**Supplementary Note 2**

Assuming that the thickness of the coil is $h$, and the diameter of the copper wire that makes up the coil is $d$, the coil diameter is $D_{1}$ and $D_{2}$, respectively. The number of turns for the two coils is

$N=\frac{D_{1}h}{2d^{2}}$ (S17)

$n=\frac{D_{2}h}{2d^{2}}$ (S18)

In terms of structural parameters, $D_{2}$ is twice the size of $D_{1}$, resulting in the turns ratio $N/n$ is 2:1. When considering a magnetic multiplier with a transmission ratio of 1:4, the number of small coils on the low-speed rotor is four times the number of large coils on the high-speed rotor. Therefore, the total turns ratio between the low-speed rotor and high-speed rotor is 1/2.

According to the electromagnetic induction law,

$E=n_{c}S\frac{\Delta B}{\Delta t}$ (S19)

where $n_{c}$ is the number of coils turns, S is the area of the coil and E is the induced electromotive force. Even if the magnets on the low-speed rotor and high-speed rotor have the same value of $\frac{\Delta B}{\Delta t}$, the induced electromotive force of the high-speed rotor is twice that of the low-speed rotor. Above all, it is proved that using a high-speed rotor to form the EMG results in better output performance.


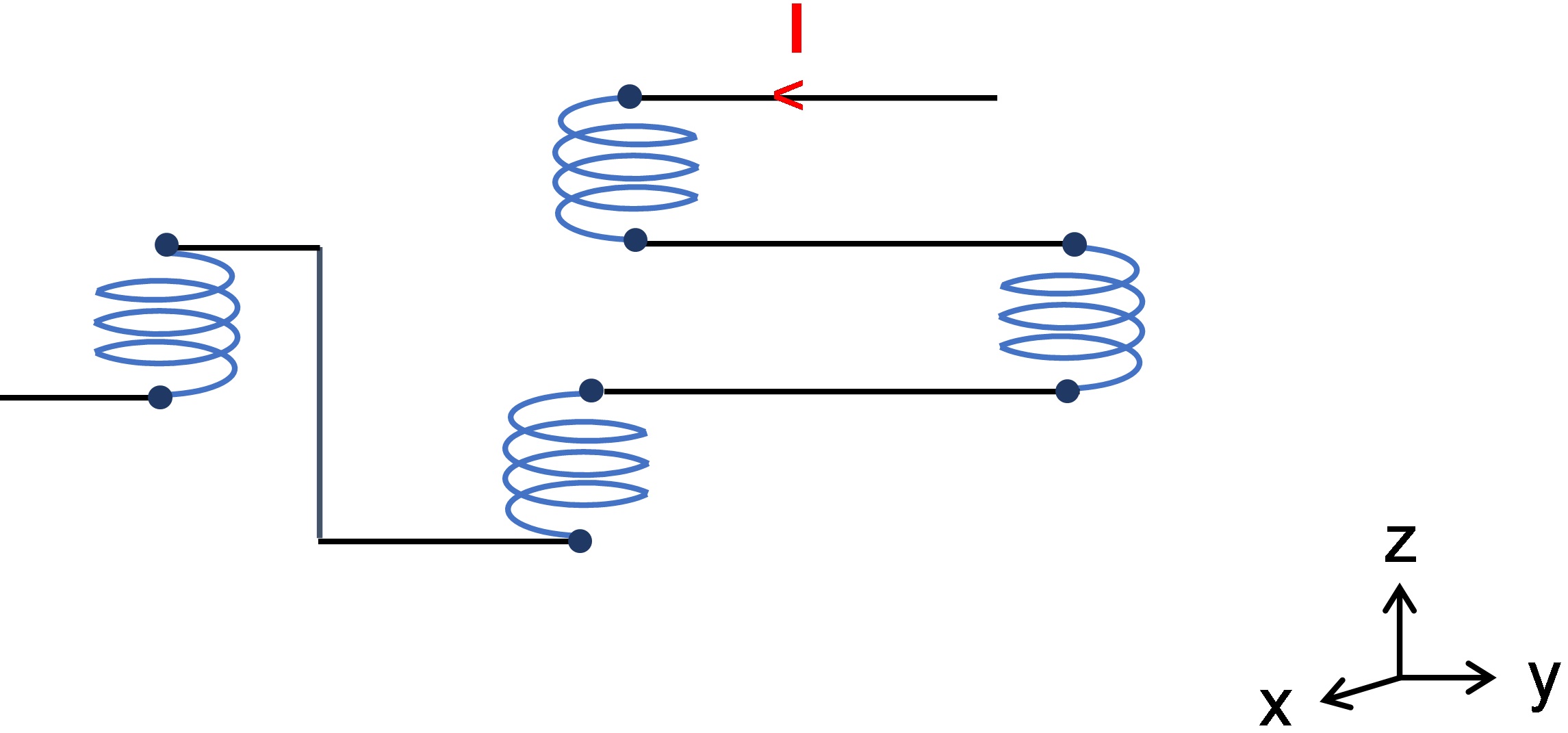


**Fig. S1.** Schematic diagram of coil’s connection in EMG.


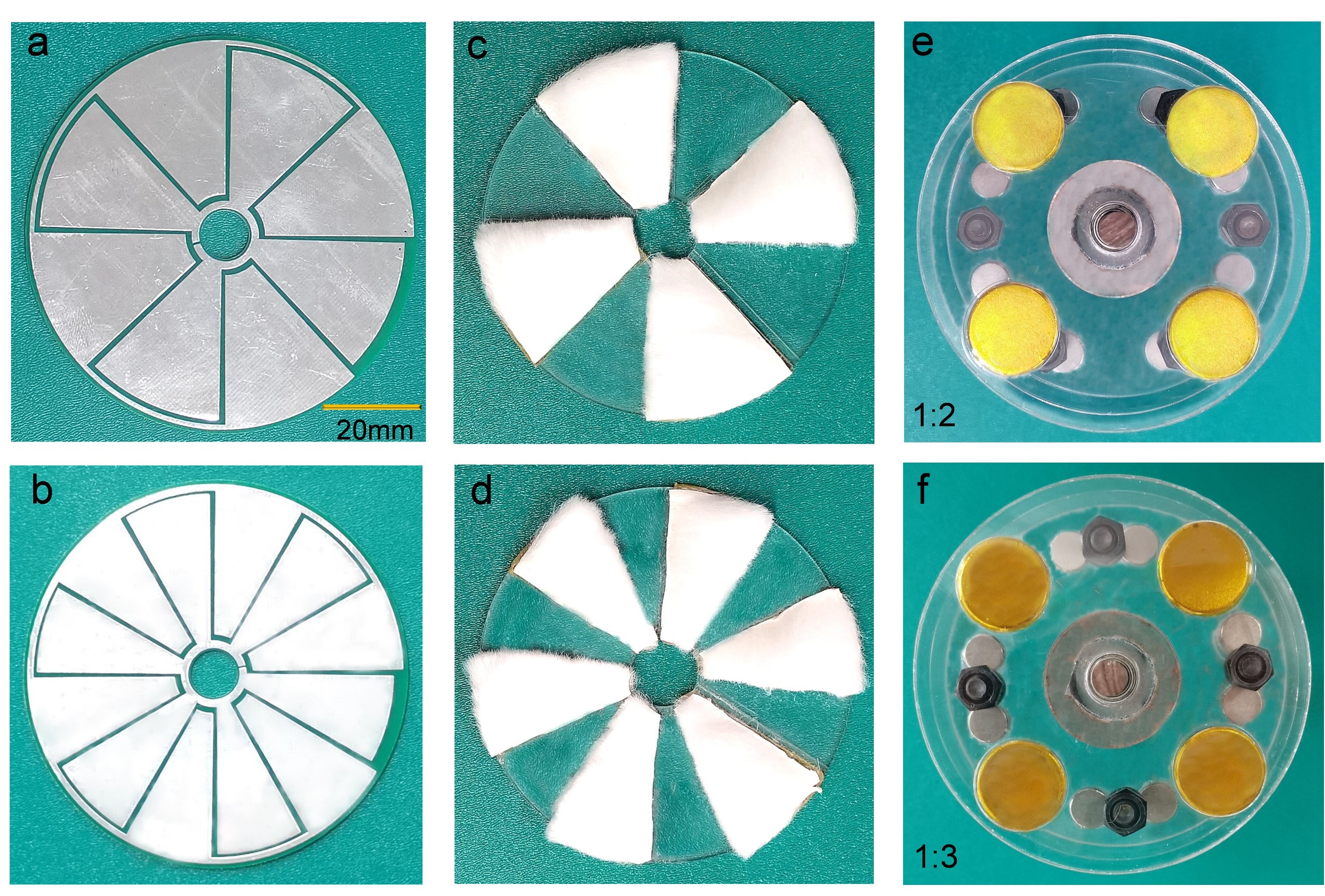


**Fig. S2**. (a)-(b) Photographs of 45°, 30° grating electrode. (c)-(d) Photographs of 45°, 30° rabbit fur tribo-layer. (e)-(f) Photographs of magnetic multiplier with transmission ratios of 1:2 and 1:3.


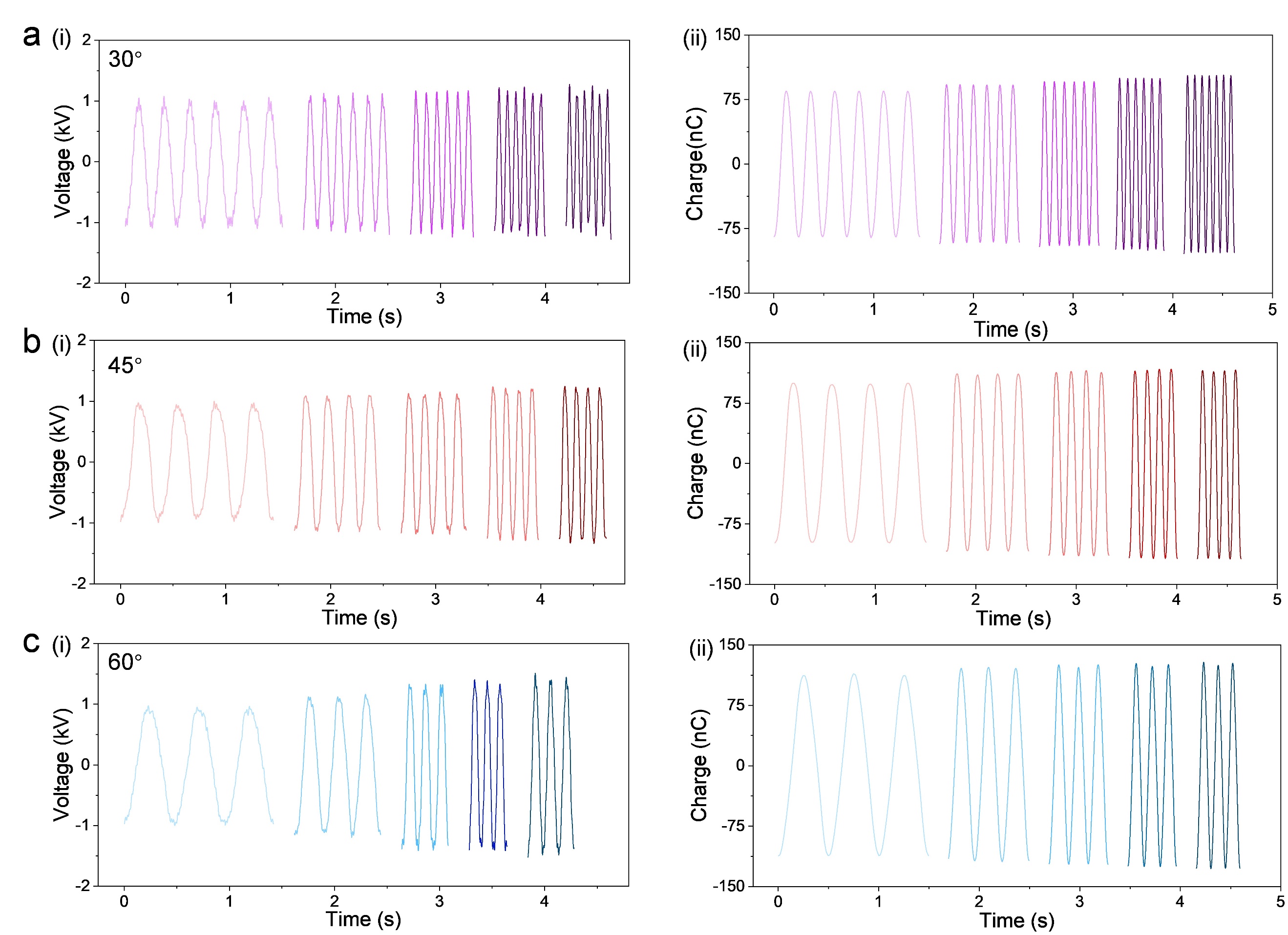


**Fig. S3**. Transferred charge and open-circuit voltage curves of 30°, 45°, 60° grating electrode obtained in one cycle at different frequencies (0.67 Hz, 1.33 Hz, 2 Hz, 2.67 Hz, 3.33 Hz).


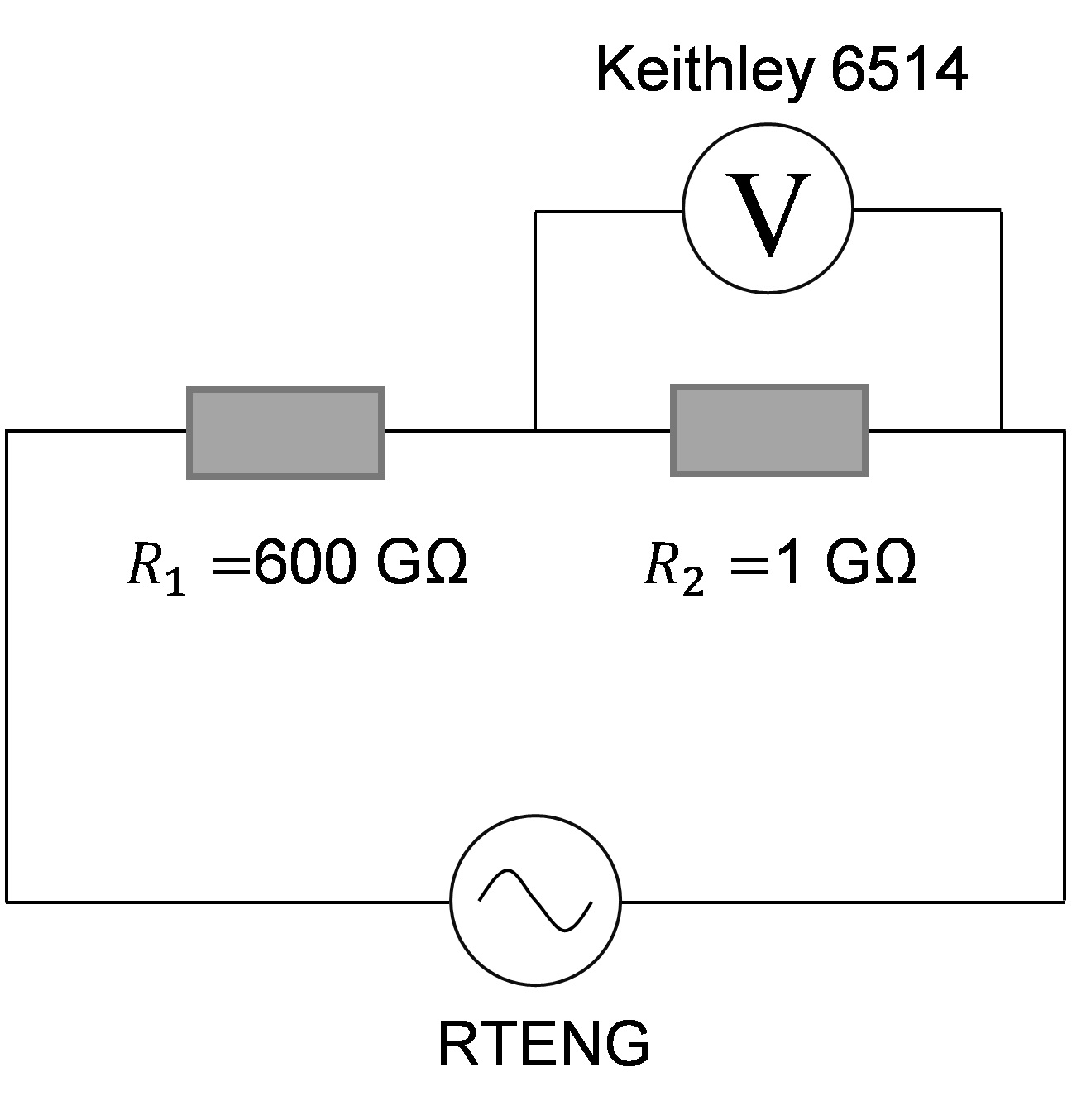


**Fig. S4**. Circuit diagram of the voltage division method.


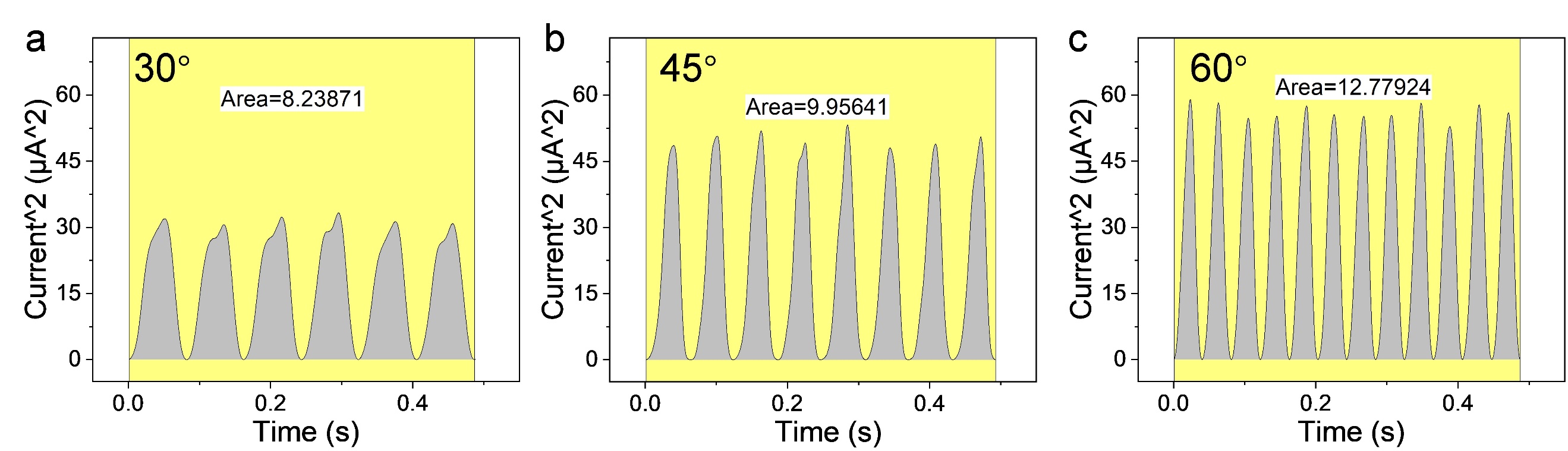


**Fig. S5**. Integral curve of 30°, 45°, 60° grating electrode obtained in one cycle.


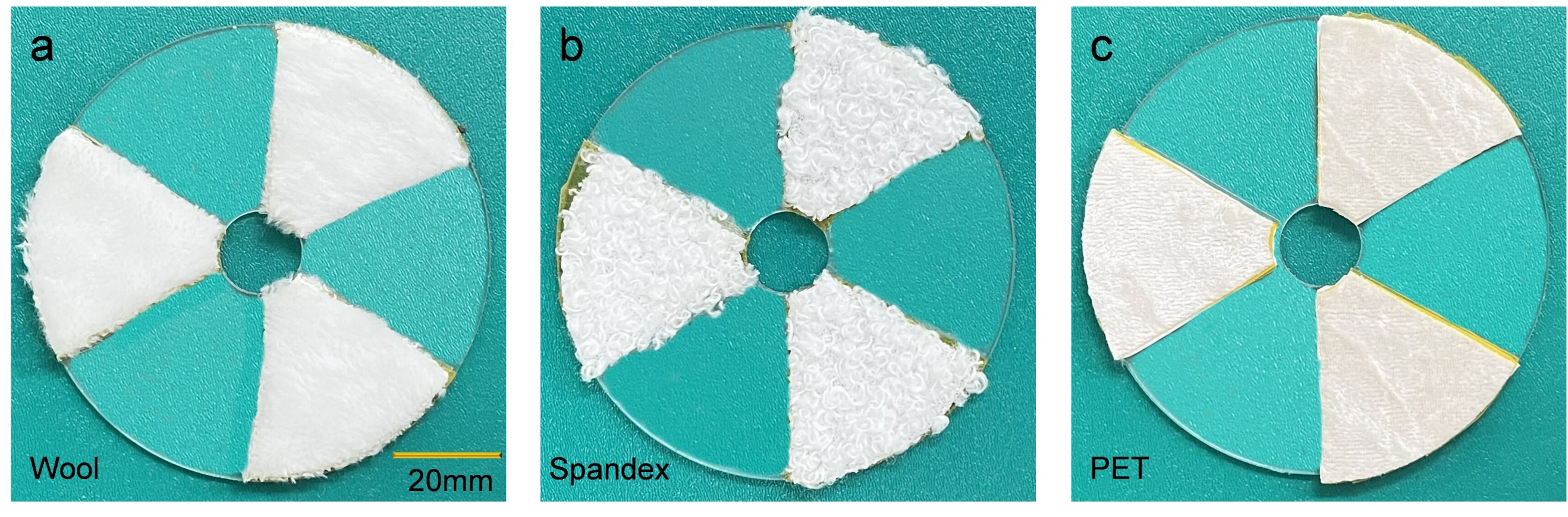


**Fig S6**. Photographs of Wool, Spandex, PET corresponding to 60° grating electrode.


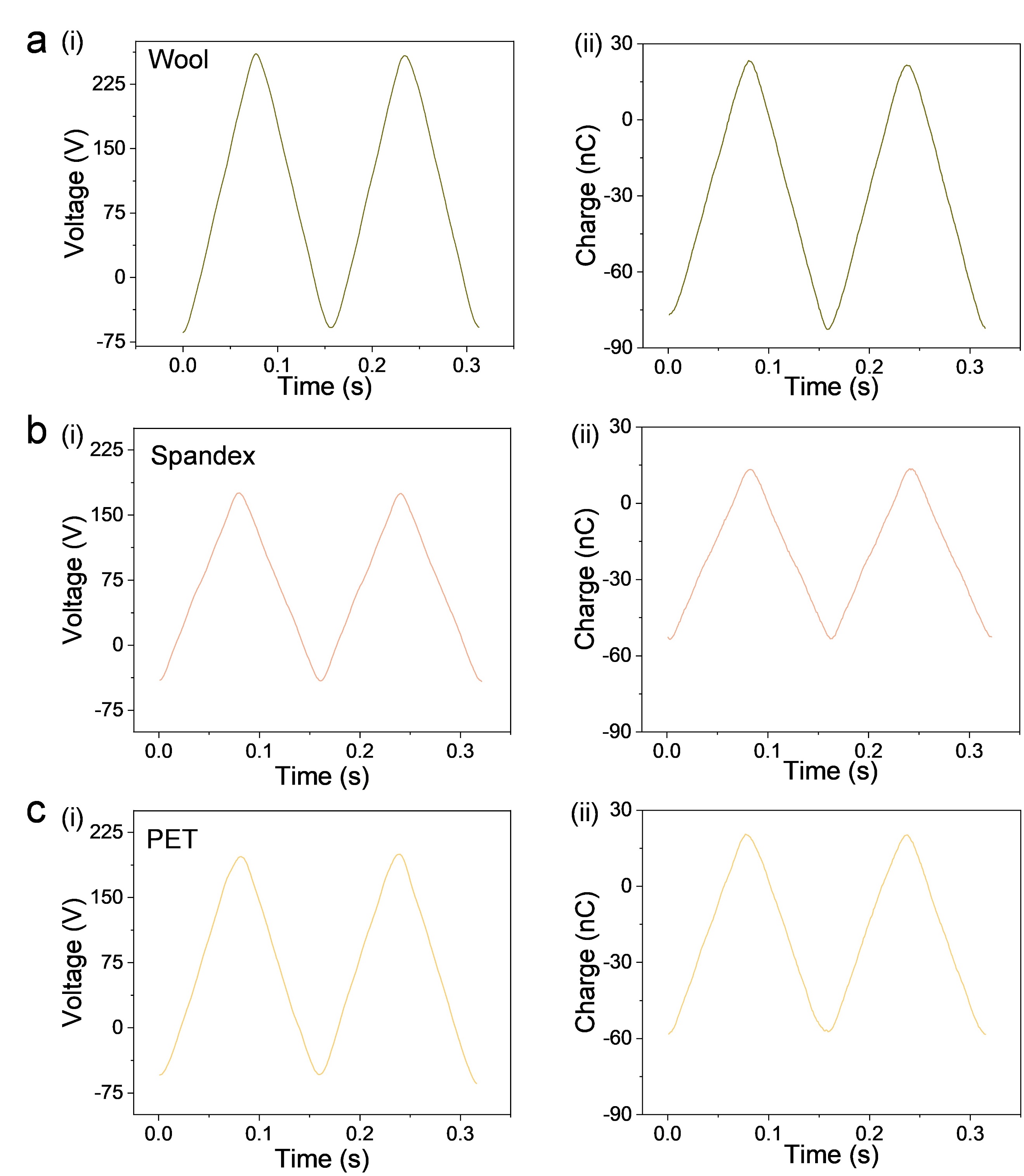


**Fig. S7**. (a)-(c) Open-circuit voltage and transferred charge curves of the Wool, Spandex, PET based TENG.


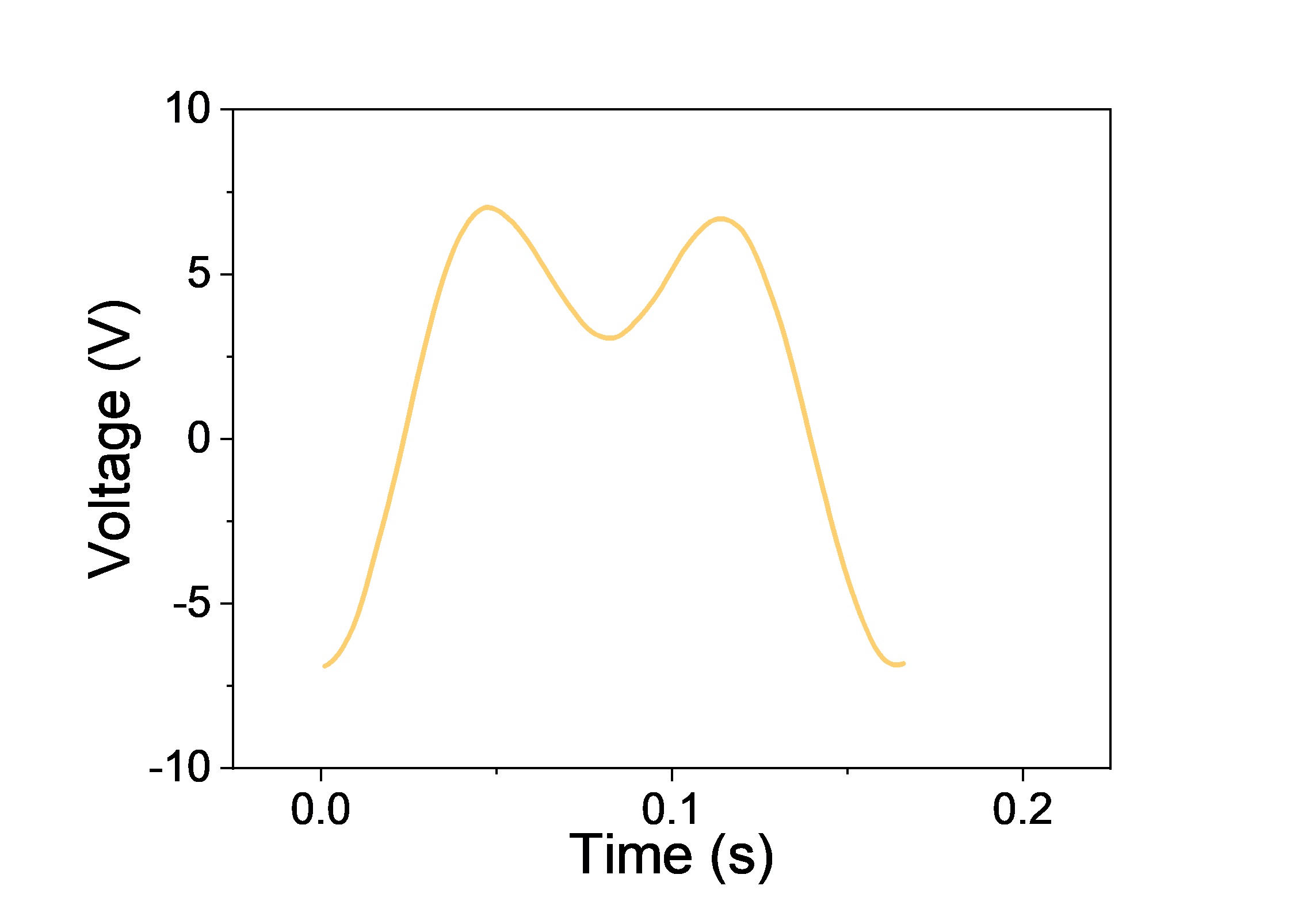


**Fig. S8**. Open-circuit voltage curves of EMG without magnetic multiplier (transmission ratio 1:1).


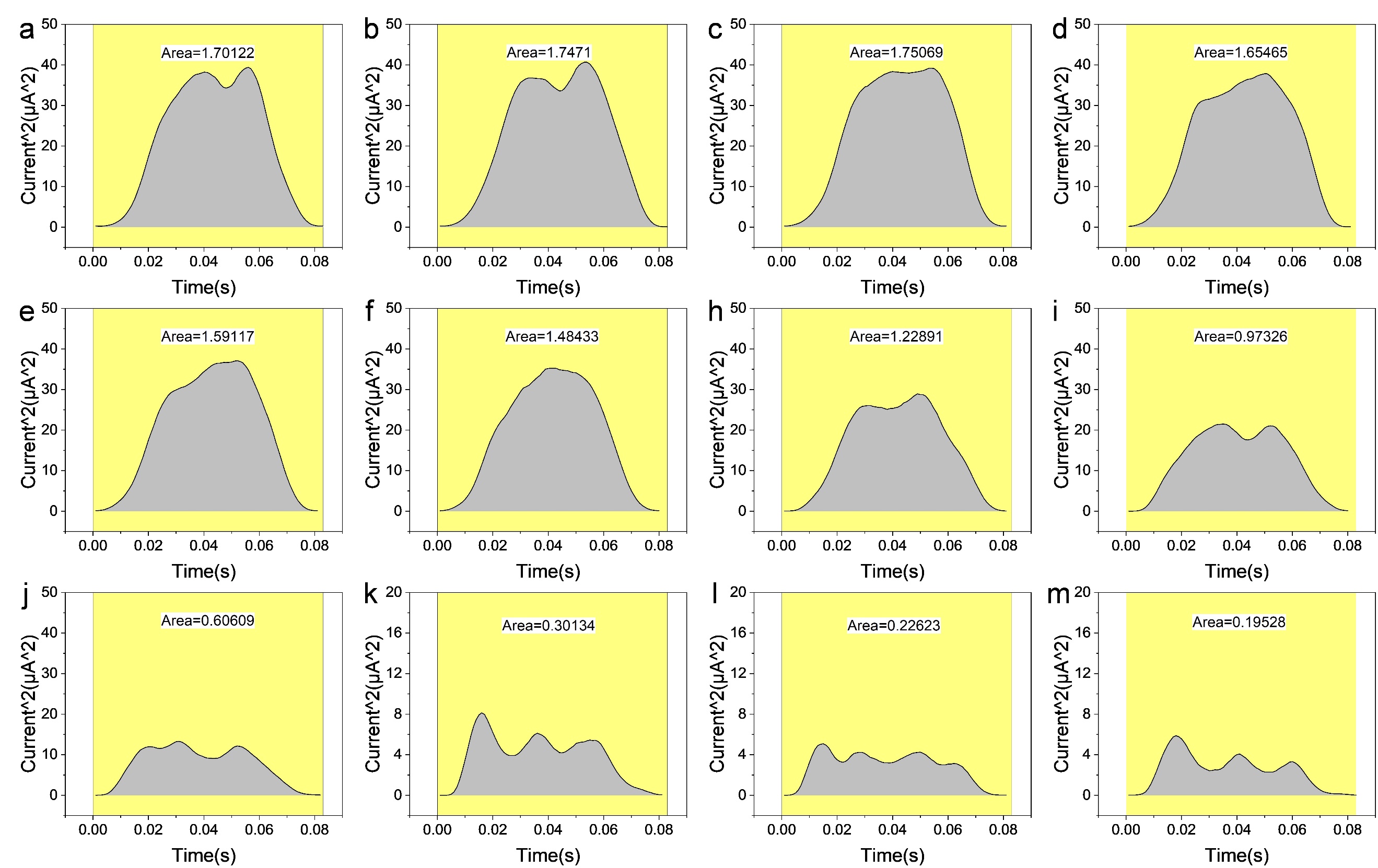


**Fig. S9**. Integral curve of series external resistors (1, 10, 50, 100, 150, 200, 250, 300, 400, 600, 700, 750 MΩ).
